# Management of Grossly Decayed Mandibular Molar with Different Designs of Split Cast Post and Core

**DOI:** 10.1155/2016/2976941

**Published:** 2016-04-10

**Authors:** Rashmi Bansal, Nakul Mehrotra, Priyanka Chowdhary, Anuraag Gurtu

**Affiliations:** Department of Conservative Dentistry & Endodontics, Institute of Dental Sciences, Bareilly, India

## Abstract

Mandibular molar with extensive loss of tooth structure, especially where no cavity wall is remaining, and insertion of posts in both the roots appear necessary so as to achieve proper retention for the core material. A single unit metal casting with two posts, one in the mesial root and the other in the distal divergent root, is difficult to fabricate due to difference in the path of insertion of the two posts. Multisection post and core or single cast post and core with auxiliary post can be an effective design to manage grossly decayed mandibular molars.

## 1. Introduction

Comprehensive treatment plan is required before the start of endodontic treatment. When the decision is made to treat the teeth endodontically consideration must be given to the placement of the subsequent restoration. Endodontically treated posterior teeth are often mutilated due to caries and access requirement, sometimes to the extent that all the walls of coronal structure are missing and only the radicular portion is present. In such cases if ferrule is available and coronal retention core buildup is not sufficient then intraradicular retention may be used by custom made post and core which replaces any lost coronal tooth structure [1]. Relatively long post with circular cross sections provides good retention and support in anterior teeth but should be avoided in posterior teeth, which often have curved roots and elliptical or ribbon shaped canals. For these teeth, retention is better provided by two or more relatively short posts in divergent canals [2]. This can be achieved by multisection post and core with each section having its own path of withdrawal or single piece post and core with a separate auxiliary post.

This paper presents two case reports on post endodontic management of badly mutilated mandibular molars: one with two-section post and core and one with single post and core with separate auxiliary post. A review of custom post and core in posterior teeth is listed in Table 1 [3–6].

## 2. Case Presentation

**Case 1 (multisection post and core).**

**Figure 1:**
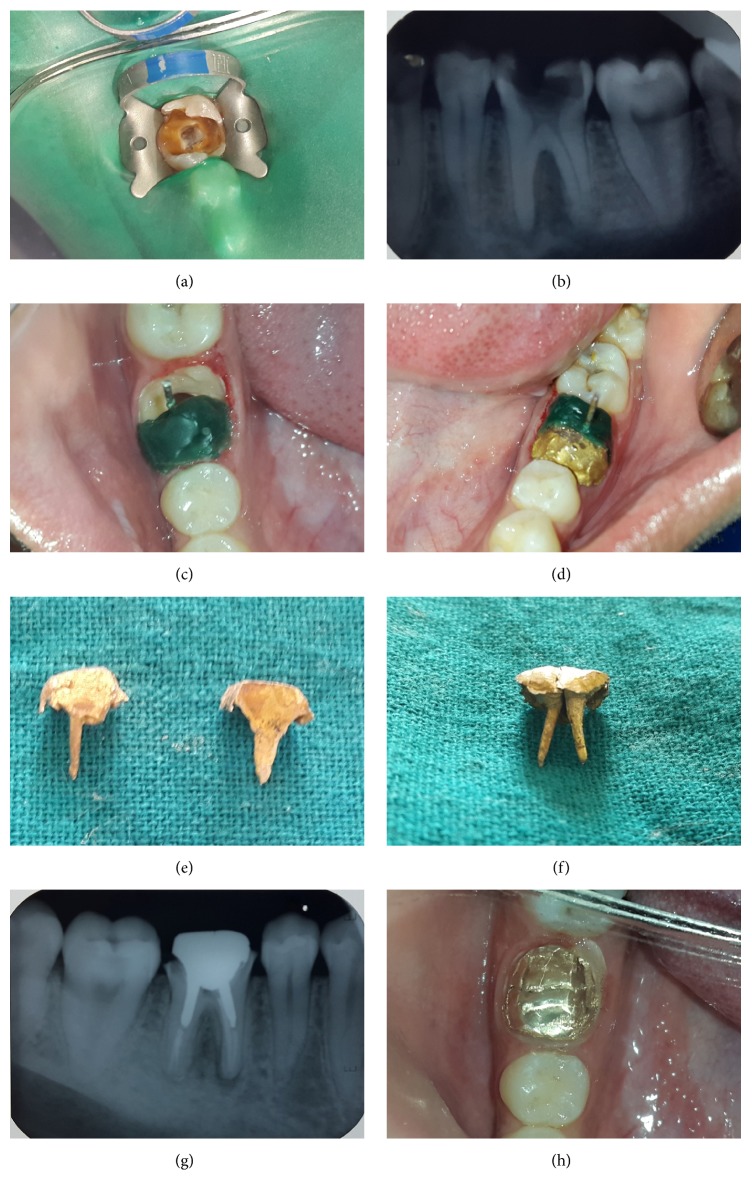
(a) Preoperative intraoral photograph showing grossly carious tooth. (b) Preoperative IOPAR showing grossly decayed molar with roots showing parallel configuration. (c) Fabrication of wax pattern in mesiolingual canal involving mesial half of the tooth. (d) Wax pattern fabrication of the distal half with mesial post and core in place. (e) Mesial and distal sections of split cast post and core. (f) Assembly of mesial and distal sections of split cast post and core. (g) IOPAR of luted and finished split cast post and core. (h) Luted and finished split cast post and core.

A 21-year-old male patient reported to the Department of Conservative Dentistry and Endodontics, Institute of Dental Sciences Bareilly, with the chief complaint of pain in lower left tooth region. Extraoral examination revealed no significant findings. Intraoral examination revealed grossly decayed 36 (Figure 1(a)). The tooth was not tender on percussion. The tooth was not mobile and nonresponsive to any pulp sensitivity tests. Intraoral sinus tract was present on the buccal aspect in left quadrant. Path of this sinus tract was traced with gutta percha using periapical radiograph. On radiographic examination, radiolucency was observed involving both the mesial and distal root of 36 (Figure 1(b)).

Chronic periapical abscess was diagnosed. A comprehensive treatment plan was made consisting of two phases: endodontic phase and restorative phase.

### 2.1. Endodontic Phase

After excavation of caries unsupported tooth structure was removed. Access cavity was refined and working length was established (distal: 13 mm, mesiobuccal and mesiolingual: 11 mm). Biomechanical preparation was completed by Mtwo files up to 6% taper number 25. During preparation canals were irrigated with normal saline (0.9% W/V) and metronidazole (0.5% W/V). A final rinse with 2% chlorhexidine solution was done after completion of biomechanical preparation. Triple antibiotic paste (ciprofloxacin 200 mg, metronidazole 500 mg, and minocycline 100 mg) was placed in the canal and patient was recalled after two weeks.

At second appointment patient was asymptomatic, and sinus tract was healed so obturation was completed with 6% taper number 25 single cone gutta percha using AH plus as a sealer. Patient was recalled after three weeks; there were no clinical signs and symptoms. Restorative phase was planned.

### 2.2. Restorative Phase

Using peeso reamer numbers 1–3 (1.1 mm diameter) post space of length 5 mm (leaving 6 mm of gutta percha apically) was prepared in the mesiobuccal canal taking care that at least minimum of 1 mm of dentin remains around the canal. Similarly post space was prepared in the distal canal using peeso reamer numbers 1–4 (1.3 mm diameter) of length 6 mm (leaving 7 mm of gutta percha apically).

Following this ferrule preparation was completed. Wax pattern of post in mesiobuccal canal with part of its core was prepared. Any undercut adjacent to another half was removed (Figure 1(c)). Casting of the mesial portion was done and the fit was checked. Direct wax pattern of the distal section of post and core was prepared with the casting of mesial section of post and core in place (Figure 1(d)). Distal section wax pattern was casted and fit was checked by keeping mesial casting in place. Dovetail to interlock the mesial and distal section was not prepared as final buildup was to be held together by the fixed cast restoration. Both the castings were luted with type II GIC (Figure 1(g)). Core preparation was finished for all metal crown. Rubber base impression was taken and was temporized for 3 weeks. All metal crown was fabricated and cemented in the next appointment. At 1-year clinical follow-up, the prosthesis exhibited no evidence of failure and the patient was satisfied with the function and esthetics.

**Case 2 (single piece core with auxiliary post).**

**Figure 2:**
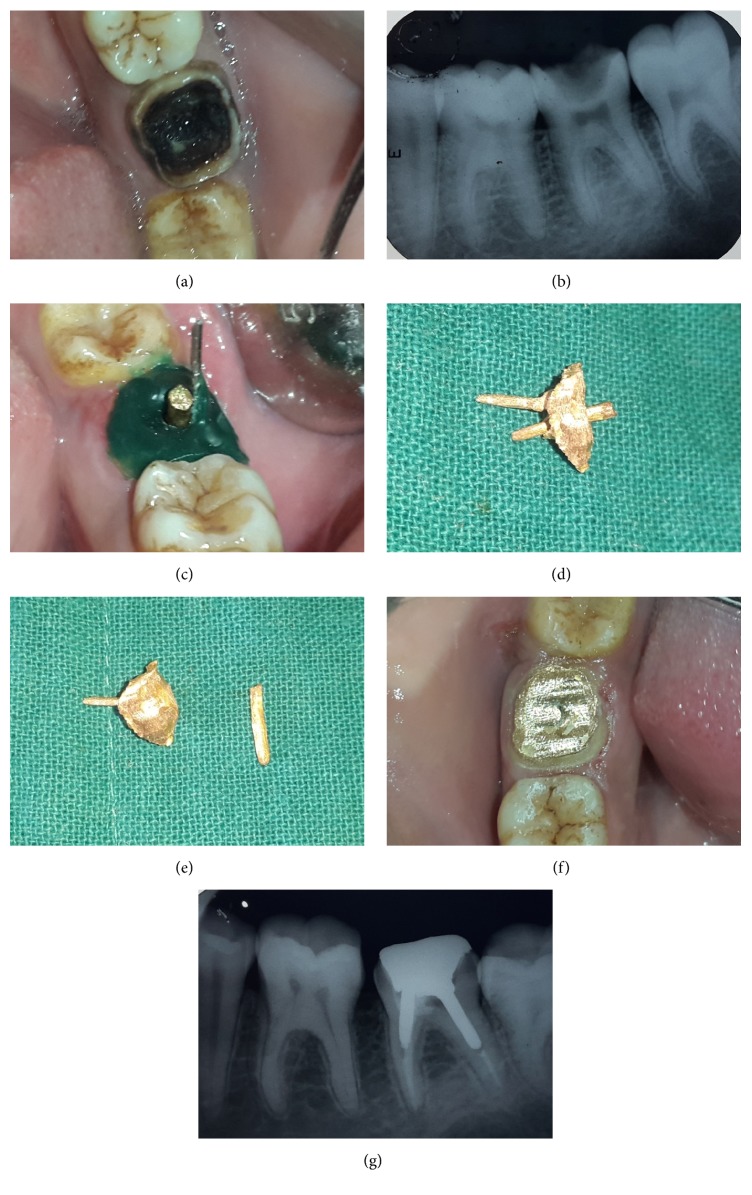
(a) Preoperative intraoral photograph showing grossly decayed molar. (b) Preoperative IOPAR showing grossly decayed molar with divergent root configuration. (c) Wax pattern fabrication of mesiolingual canal and core with distal casting in place. (d) Assembly of mesial and distal sections of split cast post and core. (e) Mesial and distal sections of split post and core. (f) Luted and finished split cast post and core. (g) IOPAR of luted and finished split cast post and core.

A 23-year-old male patient reported to the Department of Conservative Dentistry and Endodontics, with the chief complaint of decayed tooth in lower left tooth region. Extraoral examination revealed no significant findings. On intraoral examination 37 was grossly decayed (Figure 2(a)) and tender on percussion. Intraoral periapical radiograph revealed deep caries involving pulp space with no periapical changes (Figure 2(b)). There was more divergence between mesial and distal root as compared to Case 1. The tooth was nonresponsive to any pulp sensitivity tests. Endodontic therapy was planned for the tooth followed by post and core to rehabilitate the occlusal portion.

### 2.3. Endodontic Phase

Since there was no periapical change a single sitting root canal treatment was planned which was commenced by excavating all the caries and refining the access preparation. Working length was determined (distal: 13 mm, mesiobuccal and mesiolingual: 11 mm). Biomechanical preparation with Mtwo rotary files was completed up to 6% taper number 25 with concomitant irrigation using metronidazole (0.5% W/V) and normal saline (0.9% W/V). Single cone obturation was completed with 6% gutta percha number 25 using AH plus as a sealer. Patient was recalled after 1 week for restorative phase.

### 2.4. Restorative Phase

Since the divergence between the mesial and distal root was more a single core with small post in mesial root and a separate auxiliary post in distal root was planned.

Using pesso reamer numbers 1–3 (1.1 mm diameter) post space of length 4 mm (leaving 7 mm of gutta percha apically) was prepared in the mesiobuccal canal, taking care that at least minimum of 1 mm of dentin remains around the canal. Similarly post space was prepared in the distal canal using pesso reamer numbers 1–4 (1.3 diameter) of length 6 mm (leaving 7 mm of gutta percha apically).

Ferrule preparation was completed followed by the auxiliary post preparation in distal canal and single post with core preparation in the mesial canal.

### 2.5. Auxiliary Post Preparation

K file number 40 was used and its handle was removed. Green inlay wax was added to the file and impression of the distal canal was taken for auxiliary post. Post length should extend coronally beyond the actual preparation. Then impression was removed and reseated back into the canal several times while it was still soft; it was then invested and casted.

### 2.6. Single Core with Single Auxiliary Post

Wax pattern of short post in mesiobuccal canal and single core was prepared with casted auxiliary distal post in place (Figure 2(c)).

Auxiliary post was gripped with forceps and removed. Wax pattern of single short post with core was removed and casting was done. The hole for the auxiliary post was refined with the appropriate twist drill. The casting of core with post was checked with the auxiliary post through the hole into the canal. During luting of the castings, the single core and post of the mesial root was luted first followed by the immediate sliding of the distal auxiliary post through the hole in the core which was held by locking tweezer for fast and comfortable insertion (Figure 2(f)). The finishing and refining of the axial walls were done after 10 mins so that the cement is fully set (Figure 2(f)).

## 3. Discussion

The mandibular molars in the two cases presented were not having sufficient coronal tooth structure to provide retention for crown. More conservative approach was planned instead of extraction of the teeth followed by implant or fixed partial denture. Decision for cast post and core was taken as fiber or prefabricated metal post with GIC, composite, or amalgam core could have increased the chances of failure at the interface of post and core. Single cast post and core in largest and straightest canal was also not considered as single post in the distal canal may lead to either rotation of the core or inadequate retention. Further a long post also increases the chances of perforation of the root leading to failure. Internal stresses are more by placing one long post as compared to two short posts.

In both the cases, decision was made to place one long post in distal canal and one short post in the mesiobuccal canal. Mesiobuccal canal was selected instead of mesiolingual canal as more amount of dentin is present at the danger zone area in mesiobuccal canal.

**Figure 3:**
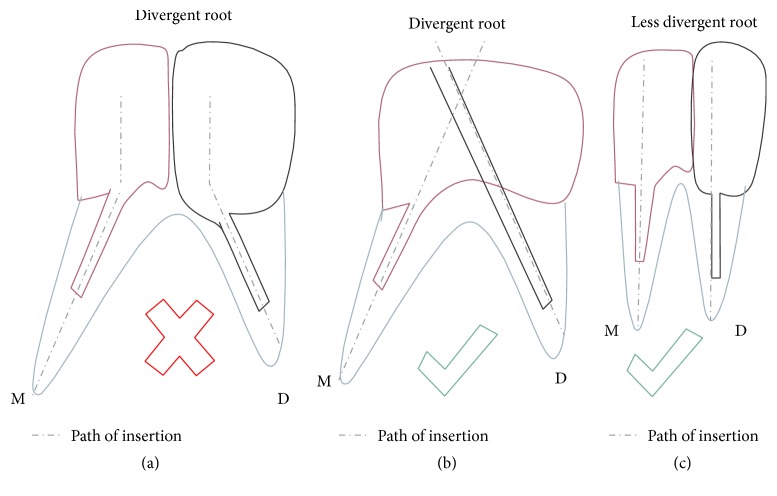
Line diagram showing different type of designs made according to the anatomy of root. In teeth with divergent roots the paths of insertion of the two posts (a) coincide with each other, so it is difficult to prepare the wax pattern. Hence in such cases the design in (b) is suggested, while in teeth with lesser divergence design in (c) is suggested.

Divergence of mesial and distal root does not allow fabrication of the two posts with core as single unit since path of withdrawal for the two posts will be different. So it was decided to prepare multisection post and core with each section having separate path of withdrawal in Case 1 (Figure 3). In Case 2 divergence between the roots was more as compared to Case 1, so long axis of post and core in each section of multisection post and core will not be in a straight line. This will cause interference during wax pattern fabrication for both the castings due to the undercut formed. Further the stresses will not be evenly distributed. Hence single post and core with auxiliary post was planned in Case 2. In both the cases two units were fabricated in separate appointments.

Preparation of dowel space within 3–5 mm of the apical seal is not considered nowadays as post length equal to the length of expected crown is thought to be sufficient. When two posts are placed in divergent roots even this length is not required and much shorter posts can provide adequate retention [7].

Multisection cast post and core reported in the literature is one in which lock and key arrangement was provided in the core of two sections [6]. This design is more technique sensitive and may require more appointments. As both the sections were resting on ferrule and are encased by full coverage crowns interlock was not required. Success in such cases was also reported in the literature previously [5].

Literature search reveals case reports in which custom cast post and core with prefabricated auxiliary post was used for restoration of badly mutilated teeth [8]. Since custom cast auxiliary post is better adapted according to canal anatomy it was also fabricated in Case 2.

*Advantages of Split Cast Post and Core.* Consider the following:

(i) Preservation of more tooth structure.
(ii) Provision of antirotation preparation.
(iii) Core retention as it is an inherent part of at least one post.
(iv) Retention of core.

*Disadvantages*. Consider the following:

(i) Placing the custom cast post and core requires additional operative and lab procedures. It is technique sensitive.
(ii) Preparing the tooth to accommodate the post requires removal of additional tooth structure.
(iii) The post can complicate or prevent future endodontic retreatment if this becomes necessary.

## 4. Conclusion

Grossly decayed mandibular molars with all walls missing can also be successfully restored by split cast post and core. Depending on the amount of divergence between mesial and distal root which affects the straight line path of withdrawal of wax pattern, multisection split post and core or single post and core with auxiliary post can be fabricated for retention of crown. Direct wax pattern technique results in precise casting. Two short posts in divergent root are sufficient to provide retention instead of one long post.

## Figures and Tables

**Table 1:** Review of literature.

S number	Author	Technique	Drawbacks
1	Bass 2002 [3]	Single post and core with auxiliary post Distal-single cast post Mesiolingual-prefabricated screw post	As the mesial canal had prefabricated post the precision was less than what could have been achieved by the custom made cast post.
2	Gogna et al. 2009 [4]	Single post and core with auxiliary post Distal-single cast post Mesiobuccal-post with core	Since mesiobuccal canal was chosen to place the post there are more chances of root perforation.
3	Kumar et al. 2013 [5]	Single post and core with auxiliary post Distal-single cast post Mesiolingual-post with core	Core was not encasing the coronal tooth structure; instead it was wedged within the coronal tooth structure.
4	Dăguci et al. 2014 [6]	Multisection post and core (lock and key arrangement) Distobuccal-single post with core Palatal-single post with core	Technique sensitive procedure.
5	Mattoo et al. 2014 [9]	Multisection post and core (lock and key arrangement) Distolingual-single post with core Mesiolingual-single post with core	Indirect pattern was used to prepare the posts that will not be as accurate.
6	Deenadayalan et al. 2015 [10]	Multisection post and core (lock and key arrangement) Distolingual-single post with core Mesiolingual-single post with core	
